# Correlation of C-reactive protein haplotypes with serum C-reactive protein level and response to anti-tumor necrosis factor therapy in UK rheumatoid arthritis patients: results from the Biologics in Rheumatoid Arthritis Genetics and Genomics Study Syndicate cohort

**DOI:** 10.1186/ar4052

**Published:** 2012-10-07

**Authors:** Darren Plant, Ibrahim Ibrahim, Mark Lunt, Stephen Eyre, Edward Flynn, Kimme L Hyrich, Ann W Morgan, Anthony G Wilson, John D Isaacs, Anne Barton

**Affiliations:** 1 Arthritis Research UK Epidemiology Unit, Manchester Academy of Health Science, University of Manchester, Oxford Road, Manchester, M13 9PT, UK; 2 NIHR Leeds Musculoskeletal Biomedical Research Unit, Chapel Allerton Hospital, The Leeds Teaching Hospitals NHS Trust, Chapeltown Road, Leeds, LS7 4SA, UK; 3 Leeds Institute of Molecular Medicine, Wellcome Trust Brenner Building, St. James's University Hospital, The Leeds Teaching Hospitals NHS Trust, Beckett Street, Leeds, LS9 7TF, UK; 4 Department of Infection and Immunity, University of Sheffield Medical School, Beech Hill Road, Sheffield, S10 2RX, UK; 5 Musculoskeletal Research Group, Institute of Cellular Medicine, Newcastle University and Newcastle upon Tyne NHS Foundation Trust, Framlington Place, Newcastle-upon-Tyne, NE2 4HH, UK; 6 Member details at end of article; 7 NIHR Manchester Musculoskeletal Biomedical Research Unit, Central Manchester NHS Foundation Trust, Manchester Academic Health Science Centre, Grafton Street, Manchester, M13 9WL, UK

## Abstract

**Introduction:**

In many European countries, restrictions exist around the prescription of anti-tumor necrosis factor (anti-TNF) treatments for rheumatoid arthritis (RA). Eligibility and response to treatment is assessed by using the disease activity score 28 (DAS28) algorithm, which incorporates one of two inflammatory markers, erythrocyte sedimentation rate (ESR) or C-reactive protein (CRP). Although DAS28-CRP provides a more reliable measure of disease activity, functional variants exist within the *CRP *gene that affect basal CRP production.

Therefore, we aimed to determine the relation between functional genetic variants at the *CRP *gene locus and levels of serum CRP in RA patients, and whether these variants, alone or in combination, are correlated with DAS28-CRP and change in DAS28-CRP after anti-TNF treatment.

**Methods:**

DNA samples from the Biologics in Rheumatoid Arthritis Genetics and Genomics Study Syndicate (BRAGGSS) were genotyped for rs1205, rs1800947, and rs3091244 by using either TaqMan or the Sequenom MassARRAY iPLEX system.

Estimated haplotypes were constructed for each sample by using the expectation maximization algorithm implemented in the haplo.stats package within the R statistical program.

CRP values were log transformed, and the association between single nucleotide polymorphisms (SNPs), haplotypes of SNPs and baseline CRP, baseline DAS28-CRP, and change in DAS28-CRP were evaluated by using linear regression in STATA v.10.

**Results:**

Baseline CRP measurements were available for 599 samples with 442 also having data 6 months after treatment with an anti-TNF. For these 442 samples, the study had > 80% power to detect a clinically meaningful difference of 0.6 DAS28 Units for an allele frequency of 5%. Estimated haplotype frequencies corresponded with previous frequencies reported in the literature. Overall, no significant association was observed between any of the markers investigated and baseline CRP levels. Further, *CRP *haplotypes did not correlate with baseline CRP (*P *= 0.593), baseline DAS28-CRP (*P *= 0.540), or change in DAS28-CRP after treatment with an anti-TNF over a 6-month period (*P *= 0.302).

**Conclusions:**

Although *CRP *genotype may influence baseline CRP levels, in patients with very active disease, no such association was found. This suggests that genetic variation at the *CRP *locus does not influence DAS28-CRP, which may continue to be used in determining eligibility for and response to anti-TNF treatment, without adjusting for *CRP *genotype.

## Introduction

Rheumatoid arthritis (RA) is a chronic, systemic, autoimmune disease that is characterized by synovial joint inflammation and, when inflammation is persistent, leads to the progressive destruction of joints [1,2].

One of the most exciting developments in the treatment of RA has been the introduction and widespread use of biologic drugs that block the tumor necrosis factor (TNF)-α pathway (anti-TNF drugs). These drugs not only reduce inflammation, but also halt radiologic damage and are responsible for spearheading a major advance in the therapeutic options available for RA patients [3]. However, limitations are associated with the use of biologic treatments, including the high costs (approximately £10,000 per patient per year), inefficacy in a significant minority of patients, and the increased risk of infection [4]. In the UK, eligibility for biologics is determined by guidance issued by the National Institute of Health and Clinical Excellence (NICE) [5]. Eligibility to start and to remain on an anti-TNF drug is determined by the 28 joint-count disease-activity score (DAS28) [6]. For example, as well as having tried and failed to respond to two previous disease-modifying antirheumatic drugs (DMARDs), individuals must also have a DAS28 > 5.1 on two separate occasions at least 1 month apart, indicating severe disease activity, before they become eligible for anti-TNF treatment.

The DAS28 can incorporate one of two inflammatory markers, erythrocyte sedimentation rate (ESR; DAS28-ESR) or C-reactive protein (CRP; DAS28-CRP) [7]. CRP is an acute-phase protein that is produced by the liver and is very sensitive to short-term changes in inflammation [8]. In contrast, the level of ESR, an indicator of long-standing, chronic inflammation, is an indirect measure of acute-phase protein and has a slow response after inflammatory stimulation or resolution [8]. ESR is also sensitive to anemia and non-acute-phase proteins such as immunoglobulin and rheumatoid factor (RF), and thus may be a better indicator of disease severity than CRP [9]. However, as the level of ESR varies with age and gender, this may confound DAS28-ESR measurements [8,9].

CRP is encoded by the CRP gene (*CRP*) on chromosome 1q21-q23. Recent genetic studies investigating this locus have shown that several single nucleotide polymorphisms (SNPs), and haplotypes of these variants, explain some of the variation in the level of serum CRP at baseline levels, as well as the variation in response to acute inflammatory stimuli [10-15]. This has led investigators to question whether genetic variants at the *CRP *locus correlate with the level of CRP in the setting of chronic inflammation [15]. Indeed, a recent study by Rhodes *et al. *(2010) observed that haplotypes of common SNPs within the *CRP *locus were correlated with CRP levels in two cohorts of patients with RA [16], although it should be noted that both cohorts had modestly active disease as determined by their median CRP levels (11 mg/L and 5 mg/L in the discovery and replication cohorts, respectively) [16]. If this correlation is also observed in patients with high CRP levels, it could have important clinical implications when assessing eligibility for anti-TNF therapy, using the DAS28-CRP [8].

The aim of the current work was first, to investigate the importance of *CRP *genetic variants in influencing CRP levels in UK patients with RA with very active disease, and second, to determine whether the genetic variants correlated with treatment response to anti-TNF drugs. Specifically, we aimed to investigate haplotypes at the *CRP *gene defined by three variants (rs1205, rs1800947 & rs3091244), which have previously been associated with levels of CRP production, to determine their association with baseline CRP levels, baseline DAS28-CRP and change in DAS28-CRP in patients with RA before and after 6 months therapy with an anti-TNF drug.

## Methods

### Subjects

DNA samples from patients included in this study were obtained from the Biologics in Rheumatoid Arthritis Genetics and Genomics Study Syndicate (BRAGGSS).

Patients eligible for the BRAGGSS cohort were initially identified through the British Society for Rheumatology Biologics Register (BSRBR). The BSRBR was launched in October 2001 and is a prospective observational study of patients with rheumatic diseases newly commenced on anti-TNF biologic therapy, who are followed up every 6 months for a period of at least 5 years [17,18]. One of the fundamental objectives of the BSRBR is to monitor patient progress, as well as the incidence of long- and short-term side effects [19,20].

The BRAGGSS cohort was developed for the study of genetic predictors of response to anti-TNF biologic therapy. Consultants at contributing centers across the UK gave permission to identify their patients from the BSRBR; eligible patients were approached by letter and invited to donate blood samples for DNA extraction and serum when they were due for a routine blood test. Samples were posted to the Arthritis Research UK Epidemiology Unit for processing, storage, and analysis. All contributing patients provided informed consent, and the study was approved by a multicenter ethics committee (COREC 04/Q1403/37) [20].

The patient-inclusion criteria for BRAGGSS are as follows: a diagnosis of RA; registration with the BSRBR; planned treatment with an anti-TNF; and Caucasian origin. In addition, a baseline and 6-month DAS28 is recorded to allow subsequent analysis.

Patients were excluded from this study if they had stopped treatment because of adverse events or reasons other than inefficacy, or after any change in their anti-TNF biologic therapy during the follow-up period [21].

### Genotyping

Genotyping for the rs1205 SNP was performed by using the Sequenom MassARRAY iPLEX system in accordance with the manufacturer's instructions [22].

DNA samples for the rs1800947 SNP and the triallelic rs3091244 SNP were genotyped by using TaqMan PCR Genotyping Assay. The primer and probe sequences for the rs3091244 assay were retrieved from the literature [23], and the rs1800947 SNP assay was available from the manufacturer. Genotyping for both SNPs was performed in accordance with the manufacturer's instructions [24].

For each marker, negative water controls were included for each experiment, and genotype cluster plots were manually reviewed. In addition, SNPs were assessed for deviation from Hardy-Weinberg Expectation (HWE).

### Haplotype inference

Haplotypes were estimated by using the expectation maximization (EM) algorithm implemented in the haplo.stats package within the R statistical environment [25]. This software supports the inclusion of SNPs with more than two alleles (that is, rs3091244).

### Statistics

Baseline levels of CRP in the cohort studied did not follow a normal distribution and were positively skewed; thus baseline CRP values were log-transformed before analysis. The association between SNPs (and haplotypes of SNPs) and CRP was evaluated with linear regression.

For the analysis of genotypic effects at each SNP, the common allele homozygous genotype was selected as the comparison group. For SNPs in which the homozygote minor allele genotype was seen fewer than 5 times, homozygote minor and heterozygote genotypes were combined.

Haplotype effects on baseline log-transformed CRP levels were determined with linear regression. The effect of each haplotype was calculated relative to those homozygous for H5, and the geometric mean values presented are the predicted values for homozygous subjects with each of the other haplotypes.

Analyses were repeated, adjusting for baseline ESR, a marker of background inflammation, independent of the influence of functional variation at the *CRP *gene. As baseline ESR levels did not follow a normal distribution and were positively skewed, ESR was square-root transformed before analysis. The relation between the transformed CRP and ESR was assessed and found to be linear. After regression analysis, baseline CRP values were derived by back-transforming the regression parameters and are reported as geometric mean values. Linear regression models were also used to analyze DAS28-CRP and change in DAS28-CRP.

These analyses were performed by using STATA v.10 [26].

Power calculations were performed by using Quanto (version 1.2.3) [27] under an additive model for a range of marker-allele frequencies.

## Results

Clinical response and demographic data were available for 2,767 patients, of whom 393 were ineligible for analysis: 244 stopped anti-TNF treatment for reasons other than inefficacy, 11 had no recorded information regarding a potential change in their therapy, and 138 had missing baseline or 6-month response DAS28 data.

The rs1205, rs1800947, and rs3091244 SNP markers were genotyped in 1,612 DNA samples. The genotyping success rate for rs1205, rs1800947, and rs3091244 was 93%, 96%, and 93%, respectively.

In total, 1,374 samples were successfully genotyped for all three SNPs, and baseline (pretreatment) CRP serum measurements were available for 599 (Table 1). Genotype frequencies at all three *CRP *markers conformed to HWE (Table 2).

**Table 1:** Demographic and disease characteristics

	(*n *= 599)
Age^a ^(years)	56.81 (10.84)
Disease duration^b ^(years)	12 (20-6)
Sex,^a ^F (%)	457 (76.29)
CRP at baseline, median (IQR) mg/L^b^	34 (17-63)
ESR at baseline, median (IQR) mg/L^b^	43 (27-68)
DAS28 at baseline^a^	6.36 (0.90)
Change in DAS28^a^	-2.01 (1.23)
Concurrent DMARD *n *(%)	449 (74.96)
Infliximab	272 (45.41)
Etanercept	220 (36.73)
Adalimumab	107 (17.86)

CRP, C-reactive protein; DAS28, Disease Activity Score 28; DMARD, disease-modifying antirheumatic drug; ESR, erythrocyte sedimentation rate; F, female; IQR, interquartile range; SD, standard deviation. ^a^Values are expressed as mean (SD). ^b^Values are expressed as median (IQR). All other values are *n *(%). Disease duration was measured in 598 patients; erythrocyte sedimentation rate (ESR) levels were measured in 578 patients; and change in DAS28 was measured in 442 patients.

**Table 2:** Relation of *CRP *genotypes to baseline CRP levels

SNP	HWE (p)	Genotype	Geometric mean CRP (mg/L) (SD)	*n*	*P *value
rs1205	0.60	GG	32.17 (2.75)	272	Ref.
		GA	31.51 (2.67)	270	0.81
		AA	26.39 (2.70)	57	0.17
rs1800947†	0.23	CC	31.93 (2.70)	533	Ref.
		CG	26.47 (2.76)	66	0.15
rs3091244_T	0.33‡	XX	29.09 (2.73)	278	Ref.
		XT	34.40 (2.72)	261	0.05
		TT	28.94 (2.53)	60	0.97
rs3091244_A¶		XX	31.36 (2.70)	550	Ref.
		XA	30.32 (2.68)	33	0.85

SNP, single-nucleotide polymorphism; HWE, Hardy-Weinberg Expectation; SD, standard copies of T allele. For the A allele of rs3091244: XX, no copies of A allele; XA one copy of A allele; AA two copies of A allele.

† No homozygote minor allele observed in this dataset.

‡ rs3091244_T & rs3091244_A SNPs combined to give HWE of rs3091244.

¶ Homozygote minor alleles grouped with heterozygotes.

Haplotypes were estimated for all 1,374 samples, and haplotype frequencies were in keeping with those reported in the literature [15] (Table 3). All haplotypes were estimated with > 98% accuracy (data not shown). Three individuals were removed from subsequent analyses because of the absence of one of the five common haplotypes (Table 3).

**Table 3:** *CRP *haplotype frequency, mean baseline CRP, DAS28, and change in DAS28

H1	G	C	T	0.31	34.01 (28.28, 40.91)	6.37 (6.20, 6.54)	-1.95 (-1.69, -2.21)
H2	A	C	C	0.27	29.91 (24.34, 36.78)	6.28 (6.09, 6.46)	-1.81 (-1.51, -2.01)
H3	G	C	C	0.31	31.87 (26.63, 38.14)	6.37 (6.20, 6.53)	-2.13 (-1.87, -2.38)
H4	G	C	A	0.05	30.32 (18.04, 50.96)	6.73 (6.26, 7.20)	-2.32 (-1.59, -3.06)
H5	A	G	C	0.06	21.97 (13.46, 35.86)	6.33 (5.89, 6.78)	-2.46 (-1.76, -0.15)

CI, confidence interval; CRP, C-reactive protein; DAS28, 28-joint-count disease-activity score.

For 442 individuals for whom change in DAS28-CRP data was available, the study had > 80% power (at the 5% significance threshold) to detect a clinically meaningful difference of 0.6 DAS28-CRP Units for an allele frequency of 5%.

No significant association was seen between any of the markers investigated and baseline CRP levels (Table 2). As rs3091244 is triallelic, two analyses were performed with each minor allele, T and A (Table 2). No association was observed between *CRP *haplotypes and baseline CRP (*P *= 0.593) (Table 3). Further, no association was found between *CRP *haplotypes and baseline DAS28-CRP (*P *= 0.540), or change in DAS28-CRP after 6 months treatment with anti-TNF therapy (*P *= 0.302) (Table 3). Adjustment for ESR did not qualitatively affect these findings (data not shown).

Not surprisingly, we noted a decrease in serum CRP levels within our cohort after 6 months of treatment with an anti-TNF, which approached the normal range (median CRP, 10 mg/L (IQR, 5, 26)). However, no association was seen between any of the markers investigated and CRP levels 6 months after treatment at the 5% significance threshold (data not shown). Further, no association was noted between *CRP *haplotypes and serum CRP levels 6 months after treatment (*P *= 0.17), or an association between *CRP *haplotypes and change in serum CRP levels 6 months after treatment (*P *= 0.19) (data not shown).

Finally, for the same 599 individuals for whom baseline CRP data were available, age at baseline (in years) and gender (female) were significantly associated with baseline levels of ESR (*P *= 0.001 and *P *= 0.012, respectively), baseline DAS28-ESR (*P *= 0.044 and *P *= 0.002, respectively) and change in DAS28-ESR (*P *= 0.0002 and *P *= 0.0062, respectively). However, age at baseline and gender did not significantly correlate with baseline CRP, DAS28-CRP, or change in DAS28-CRP (data not shown).

## Discussion

Recent reports have identified functional variants at the *CRP *locus that affect basal CRP production, and thus basal levels in a population can vary. This has potentially important consequences for RA patients. Eligibility for treatment with anti-TNF drugs is determined by use of the DAS28, which may incorporate CRP as a marker of acute-phase inflammation. Therefore, if genetic markers at the *CRP *locus affect CRP levels, these markers could influence treatment decisions, which may be particularly important in patients who carry the genotype that is correlated with low CRP production, as they may be less likely to meet eligibility criteria for anti-TNF therapy. The current study is the first to investigate the correlation between *CRP *functional SNP markers and response to treatment in a cohort of UK patients with very active disease.

In contrast to previous investigations, none of the SNP markers or marker haplotypes investigated at the *CRP *gene correlated with serum CRP level. A previous study by Suk Danik *et al. *(2006), observed correlation between each of the three SNPs investigated in the current study and median levels of CRP after an acute ischemic event, in which the median CRP value was 11.5 mg/L (IQR, 4.74, 27.69) [28]. That study reported that individuals carrying the rare homozygous AA genotype in rs3091244 had fivefold higher CRP levels than did those who did not [28]. In the current study, the AA genotype at rs3091244 was observed in only three individuals, thus making interpretation of between-study differences difficult. The most likely explanation for the differences seen between the current study and that of Suk Danik *et al. *(2006) is that any effect of the genetic markers on CRP levels is less important in more-active disease; in the current study of RA patients with longstanding (median, 12 years) disease, CRP levels were much higher (median CRP, 34 mg/L (IQR, 17, 63), Table 1; see Additional File 1).

A recent study by Rhodes *et al. *(2010) reported an association between *CRP *haplotypes and levels of CRP in RA patients [16]. Although the article concluded that *CRP *functional SNPs influence CRP level in patients with RA, the patients included in their investigation had modestly active disease, with median CRP levels of 11 mg/L and 5 mg/L in the discovery and replication cohorts, respectively [16].

The effect of inflammatory mediators (such as IL-6) and corresponding levels of inflammation may have a role in masking the effect of *CRP *SNPs on the level of serum CRP. As inflammation settles, levels of other inflammatory markers decrease, which could allow subtle genetic effects to be more clearly observed. However, within our cohort, although posttreatment levels of CRP approached levels (median CRP, 10 mg/L (IQR 5, 26)) in keeping with those reported by Rhodes *et al. *(2010), no significant genetic association was detected between any of the SNP markers or haplotypes investigated, and CRP levels at baseline or 6 months after treatment (data not shown). Indeed, 6 months may not be a sufficient time to detect a genetic effect if inflammation has not been completely terminated. Prospectively collected cohorts from which *CRP *measurements have been collected beyond 6 months after treatment will be needed to investigate this possibility.

Failure to detect an association may reflect inadequate power, particularly when investigating genotypic effects with low minor-allele frequency SNPs, suggesting that the current study may be underpowered to detect modest effects. However, despite not reaching statistical significance, the direction and strength of effect for some SNPs, for example, rs1205 and rs1800947, were in keeping with previously reported trends in association studies. Further, the sample size investigated had > 80% power to detect a change in DAS28 of 0.6 units, a meaningful difference at the 5% threshold. Genotyping success was > 90% for all three SNP markers, and haplotype estimation achieved > 98% accuracy. Moreover, haplotype frequencies corresponded well with those previously reported in the literature [15].

In the context of previous studies, such an observed lack of association may be influenced by 75% of individuals receiving concurrent DMARD therapy. However, despite the majority of individuals taking DMARDs, levels of CRP remained very high, indicating very active disease. Such variation in the current cohort may be partly explained by, first, the significant nonresponse rate seen in some individuals treated with DMARDs, and second, the necessity of nonresponse to DMARDs to satisfy the stringent anti-TNF eligibility criteria in the UK.

Recently, a number of other genetic variants have been shown to correlate with CRP levels in the general population [29]. In the future, it will be interesting to investigate the effect of these variants in influencing baseline and changes in CRP in RA patients receiving anti-TNF therapy, although it should be noted that the largest single effect on CRP levels is determined by variants in *CRP*. Hence, larger sample sizes will be required for future studies of these other loci.

## Conclusions

In summary, although genetic variation has been shown to influence CRP levels in the normal range, no evidence supports that such variation affects the levels of CRP in patients with very active inflammation. Certainly, we have observed from the current study that genetic variation at the *CRP *locus does not influence baseline CRP, baseline DAS28-CRP, or change in DAS28-CRP. We and others previously reported that age at baseline and gender influence both baseline DAS28 and change in DAS28 after 6 months of therapy [30,31]. In the current study, we confirm the known association of age and gender with baseline ESR levels but show no association of these factors with baseline CRP levels. Therefore, and most important, these findings strengthen the case for the use of DAS28-CRP in place of DAS28-ESR to assess eligibility for, and treatment response to anti-TNF medication in patients with active RA.

## Abbreviations

BRAGGSS: Biologics in Rheumatoid Arthritis Genetics and Genomics Study Syndicate; BSRBR: British Society for Rheumatology Biologics Register; CRP: C-reactive protein; DAS28: Disease Activity Score (28 joints); DMARDs: disease-modifying antirheumatic drugs; DNA: deoxyribonucleic acid; EM: expectation maximization; ESR: erythrocyte sedimentation rate; HWE: Hardy-Weinberg Expectation; IL-6: interleukin-6; IQR: interquartile range; NICE: National Institute of Health and Clinical Excellence; PCR: polymerase chain reaction; RA: rheumatoid arthritis; RF: rheumatoid factor; SNP: single-nucleotide polymorphism; TNF: tumor necrosis factor.

## Competing interests

All authors completed the Unified Competing Interest form at http://www.icmje.org/coi_disclosure.pdf and declare: no support from any organization for the submitted work. AB has received funding from Pfizer, Eli-Lilly, Sanofi-Aventis, Glaxo-Smith-Kline, Biogen-Idec and TcLand Expression, but not in relation to the submitted work in the previous 3 years. No other relationships or activities could appear to have influenced the submitted work.

## Authors' contributions

AB conceived the study, designed the study, designed the data-collection tools, organized the collaboration, monitored data collection, and drafted and revised the manuscript. She is guarantor. DP conceived the study, cleaned the data, performed statistical analysis, and drafted and revised the manuscript. II performed the genotyping, performed statistical analysis, and drafted and revised the manuscript. ML performed statistical analysis and drafted and revised the manuscript. SE and EF performed the genotyping and drafted and revised the manuscript. KLH, AWM, AGW, and JDI organized the collaboration, designed the study, and drafted and revised the manuscript. All authors read and approved the final manuscript for publication.

## Members of the Biologics in Rheumatoid Arthritis Genetics and Genomics Study Syndicate (BRAGGSS)

Cambridge University Hospitals NHS Foundation Trust, Addenbrookes Hospital, Prof H. Gaston.

Mid Staffordshire NHS Foundation Trust, Cannock Chase Hospital, Dr D. Mulherin, Dr T. Price, Dr T. Sheeran, Dr V. Chalam, Dr S. Baskar.

The Leeds Teaching Hospitals NHS Trust, Chapel Allerton Hospital, Prof P. Emery, Prof A. Morgan, Dr M. Buch, Dr S. Bingham.

Derby Hospitals NHS Foundation Trust, Derbyshire Royal Infirmary, Dr S. O'Reilly, Dr L. Badcock, Dr M. Regan, Dr T. Ding, Dr C. Deighton, Dr G. Summers, Dr N. Raj.

Doncaster and Bassetlaw Hospitals NHS Foundation Trust, Doncaster Royal Infirmary, Dr R. Stevens.

Peterborough and Stamford Hospitals NHS Foundation Trust, Edith Cavell Hospital, Dr N. Williams.

The Newcastle upon Tyne Hospitals NHS Trust, Freeman Hospital, Prof J. Isaacs, Dr P. Platt, Dr D. Walker, Dr L. Kay, Dr B. Griffiths, Dr W-F. Ng, Dr P. Peterson, Dr A. Lorenzi, Prof H. Foster, Dr M. Friswell, Dr B. Thompson, Dr M. Lee, Dr I. Griffiths.

University Hospital of North Staffordshire NHS Trust, Haywood Hospital (Stoke-on-Trent), Dr A. Hassell, Dr P. Dawes, Dr C. Dowson, Dr S. Kamath, Dr J. Packham, Dr M. Shadforth, Ann Brownfield.

Hereford Hospitals NHS Trust, Hereford County Hospital, Dr R. Williams.

Norfolk & Norwich University Hospital NHS Trust, Norfolk & Norwich University Hospital, Dr C. Mukhtyar.

Pennine Acute Hospitals NHS Trust, North Manchester General Hospital, Dr B. Harrison, Dr N. Snowden, Dr S. Naz.

Portsmouth Hospitals NHS Trust, Queen Alexandra Hospital, Dr J. Ledingham, Dr R. Hull, Dr F. McCrae, Dr A. Thomas, Dr S. Young Min, Dr R. Shaban, Dr E. Wong.

Gateshead Health NHS Trust, Queen Elizabeth Hospital, Dr C. Kelly, Dr C. Heycock, Dr J. Hamilton, Dr V. Saravanan.

Sheffield Teaching Hospitals NHS Trust, Royal Hallamshire Hospital, Prof G. Wilson, Prof D. Bax, Dr L. Dunkley, Dr M. Akil, Dr R. Tattersall, Dr R. Kilding, Dr S. Till, Dr J. Boulton, Dr T. Tait.

University Hospital of Morcambe Bay NHS Trust, Royal Lancaster Infirmary, Dr M. Bukhari, Dr J. Halsey, Dr L. Ottewell.

Sandwell and West Birmingham Hospitals NHS Trust, Sandwell General/City Hospital, Prof C. Buckley, Dr D. Situnayake, Dr D. Carruthers, Dr K. Grindulis, Dr F. Khatack, Dr S. Elamanchi, Dr K. Raza.

University Hospital Birmingham NHS Foundation Trust, Selly Oak Hospital, Dr A. Filer, Dr R. Jubb.

St Helens and Knowsley Hospitals NHS Trust, St Helens Hospital, Dr R. Abernathy.

South Tees Hospitals NHS Trust, The James Cook University Hospital, Middlesbrough, Dr M. Plant, Dr S. Pathare, Dr F. Clarke, Dr S. Tuck, Dr J. Fordham, Dr A. Paul.

County Durham and Darlington Acute Hospitals NHS Trust, University Hospital of North Durham, Dr M. Bridges.

Whipps Cross University Hospital NHS Trust, Whipps Cross University Hospital, Dr A. Hakim.

The West Suffolk Hospital NHS Trust, West Suffolk Hospital, Dr D. O'Reilly, Dr V. Rajagopal, Dr S. Bhagat.

Southampton University Hospital NHS Trust, Southampton General Hospital, Dr C. Edwards.

Basingstoke & North Hampshire NHS Foundation Trust, Basingstoke & North Hampshire Hospital, Dr P. Prouse, Dr R. Moitra, Dr D. Shawe.

Queen Mary's Sidcup NHS Trust, Queen Mary's Sidcup (QMS) Hospital, Dr A. Bamji.

Pennine Acute Hospitals NHS Trust, Royal Oldham Hospital, Dr P. Klimiuk.

Pennine Acute Hospitals NHS Trust, Rochdale Infirmary, Dr A. Bowden.

University Hospitals of Morecambe Bay NHS Trust, Furness Hospital, Dr W. Mitchell.

Central Manchester University Hospital NHS Foundation Trust, Manchester Royal Infirmary, Prof I. Bruce, Prof A. Barton, Dr R. Gorodkin, Dr P. Ho, Dr K. Hyrich, Dr W. Dixon.

Worcestershire Acute Hospitals NHS Trust, Worcestershire Royal Hospital, Dr A. Rai.

The Dudley Group of Hospitals NHS Foundation Trust, Russells Hall Hospital, Prof G. Kitas, Dr N. Erb, Dr R. Klocke, Dr K. Douglas, Dr A. Pace, Dr R. Sandhu, Dr A. Whallett.

Northumbria Healthcare NHS Foundation Trust, Wansbeck Hospital, Dr F. Birrell.

University Hospitals of Coventry and Warwickshire NHS Trust, University Hospital (was Walsgrave Hospital), Dr M. Allen, Dr K. Chaudhuri.

Wrightington, Wigan and Leigh Hospitals NHS Foundation Trust, Wrightington Hospital, Dr C. Chattopadhyay.

Nottingham University Hospitals NHS Trust, Nottingham Hospital, Dr J. McHale, Dr A. Jones, Dr A. Gupta, Dr I. Pande, Dr I. Gaywood, Dr P. Lanyon, Dr P. Courtney, Dr M. Doherty.

Salford Royal NHS Foundation Trust, Hope Hospital, Dr H. Chinoy, Prof T. O'Neill, Prof A. Herrick, Prof A. Jones, Dr R. Cooper, Dr R. Bucknall.

South Warwickshire General Hospital NHS Trust, Warwick Hospital, Dr C. Marguerie, Dr S. Rigby, Dr N. Dunn.

Weston Area Health NHS Trust, Weston General Hospital, Dr S. Green, Dr A. Al-Ansari, Dr S. Webber.

The Royal Bournemouth & Christchurch Hospitals NHS Foundation Trust, Christchurch Hospital, Dr N. Hopkinson, Dr C. Dunne, Dr B. Quilty.

Northern Lincolnshire and Goole Hospitals NHS Foundation Trust, Diana, Princess of Wales Hospital, Dr B. Szebenyi.

York Hospitals NHS Foundation Trust, York District Hospital, Dr M. Green, Dr M. Quinn, Dr A. Isdale, Dr A. Brown, Dr B. Saleem.

University Hospitals of Leicester NHS Trust, Leicester Royal Infirmary, Dr A. Samanta, Dr P. Sheldon, Dr W. Hassan, Dr J. Francis, Dr A. Kinder, Dr R. Neame, Dr A. Moorthy.

The Royal Wolverhampton Hospitals NHS Trust, New Cross Hospital, Dr W. Al-Allaf.

Greenpark Healthcare NHS Trust, Musgrave Park Hospital, Dr A. Taggart.

Chesterfield Royal Hospital NHS Foundation Trust, Chesterfield Royal Hospital, Dr K. Fairburn.

Trafford Healthcare NHS Trust, Trafford General Hospital, Dr F. McKenna.

Harrogate and District NHS Foundation Trust, Harrogate District Hospital, Dr M. Green, Dr A. Gough, Dr C. Lawson.

Royal National Hospital for Rheumatic Diseases NHS Foundation Trust, Bath Hospital, Dr M. Piper, Dr E. Korendowych, Dr T. Jenkinson, Dr R. Sengupta, Dr A. Bhalla, Prof N. McHugh, Debbie Bond.

Oxford Radcliffe Hospitals NHS Trust, John Radcliffe Hospital, Nuffield, Prof R. Luqmani, Prof B. Bowness, Prof P. Wordsworth, Dr J. David.

Milton Keynes Hospital NHS Foundation Trust, Milton Keynes Hospital, Dr W. Smith.

Royal Liverpool and Broadgreen University Hospitals NHS Trust, Royal Liverpool Hospital, Dr D. Mewar, Dr E. Tunn, Dr K. Nelson, Dr T. Kennedy.

Countess of Chester Hospital NHS Foundation Trust, Countess of Chester Hospital, Dr J. Nixon.

Royal Cornwall Hospitals NHS Trust, Royal Cornwall Hospital, Prof A. Woolf, Dr M. Davis, Dr D. Hutchinson, Dr A. Endean.

City Hospitals Sunderland NHS Foundation Trust, Royal Sunderland Hospital, Dr D. Coady, Dr D. Wright, Dr C. Morley, Dr G. Raftery, Dr C. Bracewell, Dr L. Kidd.

Stockport NHS Foundation Trust, Stepping Hill Hospital, Dr I. Abbas, Dr C. Filer.

Kettering General Hospital NHS Foundation Trust, Kettering General Hospital, Dr G. Kallarackal.

## Supplementary Material

Additional file 1 **Histogram and box-and-whisker plot of CRP levels**. **(A) **Histogram showing raw distribution of CRP levels in individuals (*n *= 599) included in analyses. **(B) **Box-and-whisker plot showing median baseline CRP level and interquartile range of individuals (*n *= 599) included in analyses. Click here for file
