# Supplementary figures and images for: Correlation of C-reactive protein haplotypes with serum C-reactive protein level and response to anti-tumor necrosis factor therapy in UK rheumatoid arthritis patients: results from the Biologics in Rheumatoid Arthritis Genetics and Genomics Study Syndicate cohort
Source: Arthritis Res Ther. 2012 Oct 7;14(5):R214. doi: 10.1186/ar4052 (PMC3580526; doi:10.1186/ar4052)

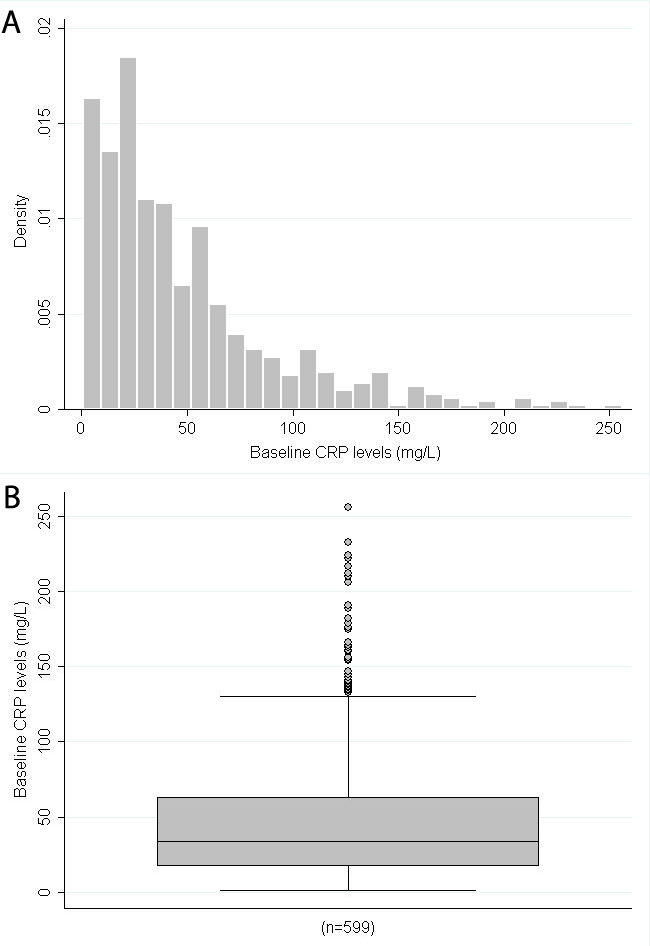

Supplement: Additional file 1 — Histogram and box-and-whisker plot of CRP levels. (A) Histogram showing raw distribution of CRP levels in individuals (n = 599) included in analyses. (B) Box-and-whisker plot showing median baseline CRP level and interquartile range of individuals (n = 599) included in analyses. [file ar4052-S1.JPEG]
